# The Royal College of British Nursing

**Published:** 1917-03-17

**Authors:** 


### THE ROYAL COLLEGE OF BRITISH NURSING.

### An Endowment Fund and Substantial Aid for Invalid Nurses.

WE are informed by the Council of the College of Nursing, Limited, that they feel they have now reached the stage at which steps should be taken to raise an endowment for the College. The arrangements necessary for making this appeal are well in hand, and the appeal will be issued shortly. It is intended that a fund shall be raised in connection with the Endowment Fund for Nurses in ill-health, or who, from no fault of their own, have fallen upon evil days.

### Members of the Irish Board.

THE following are the names of the Irish Board of the College up to date, but it is not yet complete: Miss Chisholm, Acting Superintendent, Irish Branch Q.V.J.I.N., 63 Dawson Street, Dublin; Miss Eddison, Matron, Royal City of Dublin Hospital, Dublin; Miss Egan, President Irish Matrons' Association, 8 Haddington Terrace, Kingstown, Dublin; Miss Hill, Matron, Adelaide Hospital, Dublin; Miss McGivney, Matron, Mater Misericordiæ Hospital, Dublin; Miss O'Brien, 67 Lower Leeson Street, Dublin; Miss Phelan, South Dublin Union, Dublin; Miss Reed, Ivanhoe, Lansdowne Road, Dublin; Miss Shuter, Ivanhoe, Lansdowne Road, Dublin; Andrew Beattie, Esq., D.L., 46 Fitzwilliam Square, Dublin; Charles Benson, Esq., M.D., F.R.C.S.I., 72 Lower Baggot Street, Dublin; Sir Andrew Horne, M.D., Master of National Maternity Hospital, Dublin, ex-President R.C.P.I., 94 Merrion Square; Alfred R. Parsons, Esq., M.D., F.R.C.P.I., 27 Lower Fitzwilliam Street, Dublin; Geo. Peacocke, Esq., M.D., F.R.C.P.I., 2 Fitzwilliam Square, Dublin; John Lumsden, Esq., M.D., 4 Fitzwilliam Place, Dublin; Miss Bostock, Matron, Royal Victoria Hospital, Belfast; Miss Curtin, Matron, Mater Infirmorum Hospital, Belfast; Professor Lindsay, M.D., F.R.C.P., 3 Queen's Elms, University Road, Belfast; Professor Ashley Cummins, M.D., M.Ch., 17 St. Patrick's Place, Cork; Arthur Sandford, Esq., M.D., 13 St. Patrick's Place, Cork; Miss McDowell, Superintendent of Nurses, County Infirmary, Waterford; and Miss Coffey, Matron, Barrington's Hospital, Limerick.

For "Nursing Problems in Ireland"; "Registration: a Plan of Campaign," see p. 485.
For Royal Red Cross Honours, see p. iv; Matrons' and Sisters' Appointments, p. vi.

